# Two Outbreaks of *Listeria monocytogenes* Infection, Northern Spain

**DOI:** 10.3201/eid2012.140993

**Published:** 2014-12

**Authors:** Emilio Pérez-Trallero, Carmen Zigorraga, Junkal Artieda, Miriam Alkorta, José M. Marimón

**Affiliations:** Hospital Universitario Donostia, Instituto de Investigación Sanitaria Biodonostia and Centros de Investigación Biomédica en Red, San Sebastián, Spain (E. Pérez-Trallero, M. Alkorta, J.M. Marimón);; Public Health Division of Gipuzkoa, Basque Government, San Sebastián (C. Zigorraga, J. Artieda);; University of the Basque Country Faculty of Medicine, San Sebastián (E. Pérez-Trallero)

**Keywords:** Listeria monocytogenes, outbreak, serotype 1/2b, MLST87, pregnant women, foodborne, Spain, listeriosis, bacteria

## Abstract

In the province of Gipuzkoa, Spain (≈700,000 inhabitants), 7–12 episodes of human listeriosis were recorded annually during 2009–2012. However, during January 2013–February 2014, 27 episodes were detected, including 11 pregnancy-associated cases. Fifteen cases in 2 epidemiologically unrelated outbreaks were caused by a rare type of *Listeria monocytogenes*, sequence type 87 serotype 1/2b.

Listeriosis is a bacterial zoonotic infection caused by *Listeria monocytogenes*. Most human listeriosis episodes are sporadic, but outbreaks affecting a large number of persons can distort the usual annual incidence of infection in a region. Several *L. monocytogenes* serotypes have been identified, and not all have the same capacity to infect humans; most human cases are caused by serotypes 1/2a, 1/2b, and 4b (*1*). Although the circulating serotypes have been well defined, little is known about the genetic diversity of *L. monocytogenes*. 

During January 2009–December 2012, the province of Gipuzkoa in Basque Country, northern Spain (≈700,000 inhabitants), recorded 7–12 annual episodes of listeriosis. However, during January 2013–February 2014, a total of 27 human listeriosis episodes were detected in this region. Most of the isolates identified were sequence type (ST) 87 and serotype 1/2b. To date, ST87 represents a rare ST from lineage I that had previously been reported in food (*2*,*3*) but had not been shown to cause human infections. We describe 2 epidemiologically unrelated outbreaks of listeriosis caused by ST87 that occurred at the same time in the same region. For 1 of these outbreaks, the causative agent was found in contaminated food.

## The Study

In Gipuzkoa, 27 human listeriosis episodes were reported during January 2013–February 2014. All cases produced sepsis in the patients, except 1 case that produced diarrheal disease in a 34-year-old parturient woman who had undergone a splenectomy. Eleven episodes (40.7%) occurred in pregnant or parturient women, and 8 of the children of these patients were affected: 5 newborns (4 of them premature infants) became ill, 2 pregnancies ended in miscarriage, and 1 infant was stillborn. (For this study, a pregnancy-associated episode was counted only once, whether the causative strain was isolated from mother, child, or both.) Ten cases (37.0%) occurred among the elderly (>70 years of age) and 6 (22.2%) in adults 45–60 years of age. Of the 6 patients in the 45- to 60-year age group, 3 were apparently healthy and 3 immunocompromised. Three of the 10 elderly patients died.

Human *L. monocytogenes* isolates were obtained by using routine microbiological procedures. A total of 29 human *L. monocytogenes* isolates were available for microbiological characterization: 22 from blood, 3 from placental membranes, 2 from cerebrospinal fluid, and 1 each from stool and dermal exudate. Serotypes were established by agglutination (Listeria-O-antisera, Difco; BD Diagnostics, Sparks, MD, USA) and by multiplex-PCR (*4*). The predominant serotypes identified were 1/2b (n = 17, 58.6%) and 4b (n = 8, 27.6%); 4 isolates were serotype 1/2a (13.8%).

Pulsed-field gel electrophoresis (PFGE) was performed by using the restriction endonucleases *Sma*I and *AscI* (*5*). The STs of *L. monocytogenes* isolates were determined by using the multilocus sequence typing (MLST) primers and conditions described by the Pasteur Institute (*6*). MLST showed that 16 of 17 serotype 1/2b isolates we tested were ST87; the remaining serotype 1/2b isolate was ST3. Only 2 human *L. monocytogenes* ST87 serotype 1/2b isolates, both from Japan, were listed in the Pasteur Institute MLST database (*6*). Further, PFGE showed 2 large clusters within serotype 1/2b, and epidemiologic research detected 2 main outbreaks. All listeriosis episodes during the study period that were caused by isolate types other than serotype 1/2b and ST87 were sporadic.

In the first case cluster (first outbreak), 5 episodes were detected during August–September 2013; of these, 3 were pregnancy associated. All isolates from 1 woman at 28 weeks’ gestation, 1 parturient woman and her newborn child, and 2 newborn twins (no samples from the mother were available) showed the same PFGE pattern with the restriction enzyme *Asc*I (arbitrarily named as pattern A) and were ST87 (Figure). Another 2 *L. monocytogenes* isolates from cases apparently not related to this first outbreak, isolated in January 2012 and in April 2013 from a 53-year-old man with meningitis and an 84-year-old woman, respectively, showed the same PFGE pattern.

**Figure:**
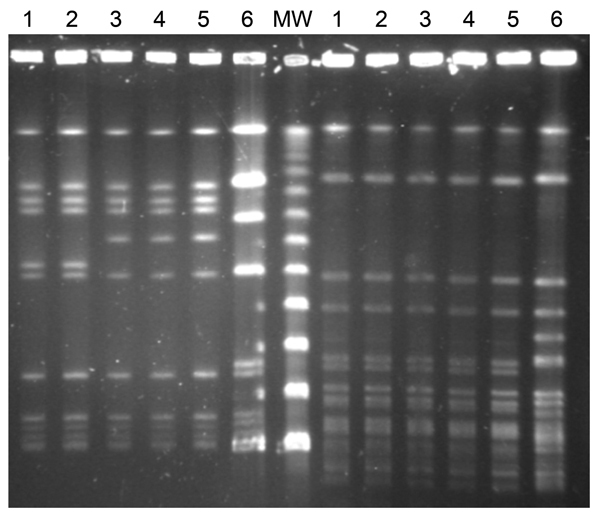
Pulsed-field gel electrophoresis patterns of 6 *Listeria monocytogenes* serotype 1/2b isolates collected from listeriosis patients in Gipuzkoa, northern Spain, during January 2013–February 2014. Left side, after *Asc*I restriction; right side, after *Sma*I restriction. Lanes 1 and 2, isolates from first outbreak (pattern A); lanes 3, 4, and 5, isolates from second outbreak (pattern B), including (lane 5) 1 isolate from a food product of foie gras; lane 6, *L. monocytogenes* sequence type 3 serotype 1/2b isolate, unrelated to outbreaks. MW, DNA molecular weight marker (50-kbp ladder).

For this first outbreak, epidemiologic interviews were conducted starting with the cases observed during September 2013. Patients were asked about their alimentary habits, focusing on consumption of raw or semicooked animal or animal derivate products. Although no specific food was identified as the possible source of the outbreak, all but 1 of the patients remembered eating cooked ham bought in the butcher’s department of a certain supermarket chain located in different villages of the province. *Listeria* spp. in food products were investigated by PCR and culture after selective enrichment (24 Listeria Enrichment Broth; Oxoid, Basingstoke, UK)). In the microbiological investigation (*7*), 1 of 6 brands of cooked ham studied grew *L. monocytogenes* at a low level, but the sample strain was not the serotype 1/2b identified in the outbreak. No *Listeria* spp. were identified in the remaining 5 brands of cooked ham studied.

In the second outbreak, 10 episodes were reported during early November 2013 through late February 2014; patients were 5 elderly persons, 4 previously healthy parturient women, and 1 parturient women who had undergone a splenectomy. No isolates could be obtained from the mother of an infected newborn and from a miscarriage. The 10 isolates available for study (1 per episode) were of the same serotype and sequence type, 1/2b and ST87, as isolates from the first outbreak, but these isolates differed in PFGE *Asc*I pattern (this pattern arbitrarily named pattern B). Isolates from these 2 outbreaks had the same PFGE pattern when the *Sma*I enzyme was used (Figure).

After the epidemiologic survey for this outbreak, we conducted microbiological analysis of several foods. A foie gras product kept by a patient in his refrigerator yielded a positive culture of *L. monocytogenes* that had the same phenotype and molecular profile as isolates from the second outbreak. The presence of the outbreak strain was confirmed in high amounts (5.2 × 10^4^ CFU/g) by several other cultures from 3 unopened samples from the same brand of foie gras. After this food source was identified as the source of this second outbreak, all patients from the first outbreak were specifically interviewed again about the consumption of this product, but none remembered having eaten it.

## Conclusions

*L. monocytogenes* infection is serious and has high fatality rates. Among the 35 persons infected in this region of Spain since 2013, a total of 6 deaths have occurred: 3 adults, 2 fetuses (miscarriages), and 1 child (stillbirth). Without rapid case detection and early treatment, the lethality of these infections could have been much higher.

*L. monocytogenes* infections mainly affect elderly persons, pregnant women, newborns, and immunocompromised adults (*8*–*10*). Humans are usually infected after eating contaminated food, although the source of the infection is infrequently detected in sporadic cases. For outbreaks, after the epidemiologic survey, a food is usually implicated as the source of infection, but it is not always possible to obtain microbiological confirmation (*11*). In this study, the strain (same phenotype and genotype) that caused the second outbreak was obtained from a recently consumed food in the home and in several unopened samples from the same batch of food. However, this source could not be identified as related to the first outbreak.

Pregnant women were frequently infected during these outbreaks; epidemic clones infected 8 pregnant women and their offspring. However, during 2012 and 2013, serotype 4b isolates of different genotypes also infected 5 pregnant women, resulting in 1 miscarriage, 3 premature newborns, and 1 stillborn infant and 1 premature newborn in a twin pregnancy.

In summary, we defined 2 epidemiologically unrelated outbreaks of listeriosis caused by a rare type of *L. monocytogenes* that occurred at the same time in a small region of Spain. Management of this frequently fatal disease requires careful investigation of the source of infection to stop its spread and prompt treatment of infected persons to prevent severe illness and death.
